# ABCC11 Earwax Trait and Genotype Are Suitable Tools for Introductory Labs to Learn Genetics and Molecular Techniques

**DOI:** 10.1002/bmb.70010

**Published:** 2025-08-13

**Authors:** Tohru Ohta, Rie Takai, Akiko Yoshida, Durga Paudel, Sarita Giri, Takao Kitagawa, Toshiya Arakawa, Yasuhiro Kuramitsu, Tomoharu Tokutomi

**Affiliations:** ^1^ Advanced Research Promotion Center Health Sciences University of Hokkaido Tobetsu Japan; ^2^ Division of Biochemistry Department of Oral Biology Health Sciences University of Hokkaido Tobetsu Japan; ^3^ Department of Clinical Genetics, School of Medicine Iwate Medical University Morioka Japan; ^4^ Department of Pediatrics Kawasaki Medical School Kurashiki Japan

**Keywords:** ABCC11, genetic education, hands‐on learning, high school science, mendelian inheritance

## Abstract

Professional experiments in genetic research usually start in a class at university. However, interest in genetic research techniques from an early age is essential. We have continuously performed a short genetic experimental course for high school students using a simple molecular experiment and computer‐based learning for Mendelian inheritance. We utilized the phenotype–genotype association of ABCC11 polymorphism (rs17822931), in which the A allele at rs17822931 of ABCC11 is the recessive genotype for the dry earwax phenotype. Conversely, the G allele is the dominant genotype for the wet earwax phenotype. The phenotype is primarily determined by a single‐nucleotide polymorphism (SNP), rs17822931, with the A or G allele, which has global prevalence with few exceptions. The A or G allele is easily typed using the DdeI restriction enzyme. The students experienced molecular techniques and created computer‐equipped pedigree charts using the software “f‐tree.” The earwax trait is an excellent tool for genetic education to understand Mendelian inheritance, genotype–phenotype association, PCR cloning, and restriction enzyme digestion, and it is suitable for discussing the historical and geographical migration of the ancient Mongolian people. After the short course, a survey showed that 81% of students were satisfied, including 71% of very satisfied students.

## 1 Introduction

Molecular genetics technology is progressing in many fields, including medical, industrial biology, and academic research. Advanced experiments for molecular genetics research usually start in a class at university. However, interest in molecular genetics experiments should be encouraged from an early age [1, 2, 3, 4, 5]. Scientific education for young students is usually performed in classroom lectures using textbooks or computer‐based learning using special software or audio‐visual learning [6, 7, 8]. In addition to traditional educational methods, assessing genetic knowledge and attitudes is critical for understanding how students interact with and comprehend genetic information. Yoshida et al. [9] developed and validated a Japanese version of the International Genetics Literacy and Attitudes Survey, a valuable tool for gauging and improving genetic literacy in educational settings. Performing actual experiments is ideal for learning molecular genetics and molecular biology. Some advanced high schools perform experiments using web‐based or wet experiment methods [10, 11]; however, such experiments are difficult to perform in regular high schools, owing to the cost of the material and special instruments. The ABCC11 gene, responsible for determining earwax type, offers a unique opportunity for genetic education due to its simple Mendelian inheritance pattern [12]. ABCC11, whose polymorphism is easily identifiable through basic molecular techniques, is an excellent model for illustrating fundamental genetic concepts such as dominant and recessive traits. Utilizing ABCC11 in educational settings allows students to observe the implications of genetic variation and engage with it directly. We have continuously provided high school students with a short (2‐day) genetic experimental course using a simple molecular experiment. Consequently, the students attending the genetics course have been able to molecularly clone their earwax gene and identify DNA variation for earwax phenotype to understand Mendelian inheritance traits. In addition, we also provided computer‐based learning involving students drawing a family tree of individual pedigree based on Mendelian inheritance. A previous study reported that experience‐based teaching, which utilizes alcohol tolerance phenotype as a student‐derived real‐life example, enhances understanding of genetics education [13]. More specialized experiential education includes courses introducing qPCR to quantify the expression of chemical defense genes and CRISPR training modules, but these require a long implementation period [14, 15]. The course aims to impart students with an understanding of the basic principles of Mendelian genetics by analyzing familiar Mendelian phenotypes using fundamental molecular biology techniques, thereby enabling them to interpret the inheritance patterns of their traits.

## 2 Materials and Methods

### 2.1 Participants

Participants for the genetic course were selected based on their previous performance in introductory biology courses, ensuring they had foundational knowledge relevant to genetics. Before experimentation commenced, a preparatory session was conducted to introduce fundamental molecular biology and genetics concepts, ensuring all students were adequately prepared for the interactive practical activities. The number of attendees at the course was different each year. Approximately 25 students performed the molecular experiment at the same time. However, the students were subsequently divided into groups A and B when more students attended the course. On the first day, the high school students participated in a lecture on molecular genetics to aid general understanding. Another lecture was delivered on the second day of the short course by an invited professional researcher or clinician, different each year. The summarized timeline is as follows:

Day 1

− 2 h lecture on molecular biology.
− 1 h practice for manipulation of PIPETMAN.
− 1 h harvesting of mouth mucosa cells and DNA extraction setup.
− 1 h lecture on PCR, restriction enzyme digestion, and electrophoresis.
− 1 h genomic DNA purification and PCR preparation.
− Overnight running of PCR.

Day 2

− 0.5 h restriction enzyme digestion of PCR products.
− 1.5 h special guest lecture.
− 3 h electrophoresis of digested PCR products and hands‐on activities, including onion. Genomic DNA isolation and computer‐based pedigree chart drawing.
− 1 h discussion of individual results.

### 2.2 Molecular Cloning of the Earwax Gene and Detection of the Variation

Before the course started, students' parents were required to provide written informed consent to use their children's genomic DNA. The ethics committee of the Health Science University of Hokkaido approved the study (#20‐Hg‐003).

The oral mucosa cell was obtained using an Omni Swab (Qiagen). The genomic DNA was extracted using a QIAamp DNA Micro Kit. The swab was incubated in 600 μL of ATL buffer containing a detergent to lyse the cells and nuclear membrane, with proteinase K at 56°C for 1 h. Ethanol (300 μL) was added to the supernatant liquid, which was applied to the QIAamp DNA Micro column after incubation at 70°C for 10 min in AL buffer containing guanidine hydrochloride and malic acid to denature proteins. A 40 μL solution, including genomic DNA, was obtained after rinsing with AW1 and AW2. The wash buffers AW1 and AW2 contained different concentrations of ions and ethanol to completely purify the genomic DNA bound to the silica membrane in the tube. Subsequently, 20 μL of the solution was used as a template for PCR. The PCR was performed with the TaKaRa Taq Hot Start Version (Takara) at 94°C for 2 min and subsequently at 94°C for 20 s, 58°C for 20 s, and 72°C for 30 s for 31 cycles using primers of TO297EarWaxF (5′‐TGCAAAGAGATTCCACCAGTT‐3′) and TO298EarWaxR (5′‐AAGGTCTTCATTTTCTAGACAGC‐3′) in a total concentration of 50 μL reaction buffer [16].

Furthermore, 20 μL of the PCR product was digested with 1 μL of DdeI (NEB), including 4 μL of 10× buffer, in a total concentration of 40 μL without any purification of the PCR product after PCR was completed. The agarose gel was prepared using 0.5 M Tris‐acetate‐EDTA buffer with agarose powder at a final concentration of 2.5%, similar to the standard electrophoresis procedure used for DNA analysis. Given the fragility of the agarose gel, inexperienced students were limited to applying their samples to fewer than eight wells. Finally, the DdeI‐digested PCR product was electrophoresed in the agarose gel after incubation for 3 h at 37°C.

### 2.3 Computer‐Based Learning for Pedigree Drawing

The pedigree chart, including earwax characteristics (wet, dry, unknown) as family health histories, after a brief lecture on the usage, was created using the software “f‐tree” as previously described [17]. In a computer‐equipped classroom, the instructor could check the operation of all participants.

f‐tree is free‐to‐use software and can be downloaded directly from its official website (https://www.holonic‐systems.com/f‐tree/en/). The software automatically generates medical pedigree charts based on family information and is widely used in the clinical field, particularly for genetic counseling in Japan [17].

To get started with f‐tree, users are required to follow these steps:

1. Download the software from the above link and install it on a compatible computer.
2. Using the guided interface, input family information, including traits such as earwax type.
3. Generate a pedigree chart by following the built‐in instructions or tutorials on the website.

This approach ensures that others can easily download, install, and replicate the module's activities in a comfortable setting.

### 2.4 Final Questionnaire

Finally, we determined participants' (*n* = 126 enrolled in our study between August 2019 and January 2020) satisfaction with the practical training using a questionnaire with the following levels: very satisfied, satisfied, average, dissatisfied, and very dissatisfied.

## 3 Results

### 3.1 Molecular Cloning and Genotyping

Approximately 3 h are required to isolate individual genomic DNA to manipulate PIPETMAN. The genomic DNA was amplified directly without measuring the DNA concentration. Almost all students could obtain substantial PCR products to detect bands in agarose gels with electrophoresis, owing to the simplicity of the short course training.

**FIGURE 1:**
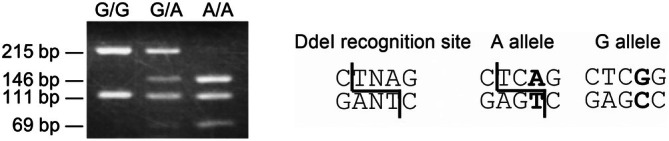
Three banding electrophoresis patterns after restriction enzyme digestion of the PCR product. A or G alleles could divide the 215 bp band into 146 bp and 69 bp bands, respectively. Additionally, the 111 bp band demonstrated positive control to confirm the effect of DdeI. The DdeI restriction enzyme recognition site and the A and G alleles nucleotide sequences are shown on the right.

**FIGURE 2:**
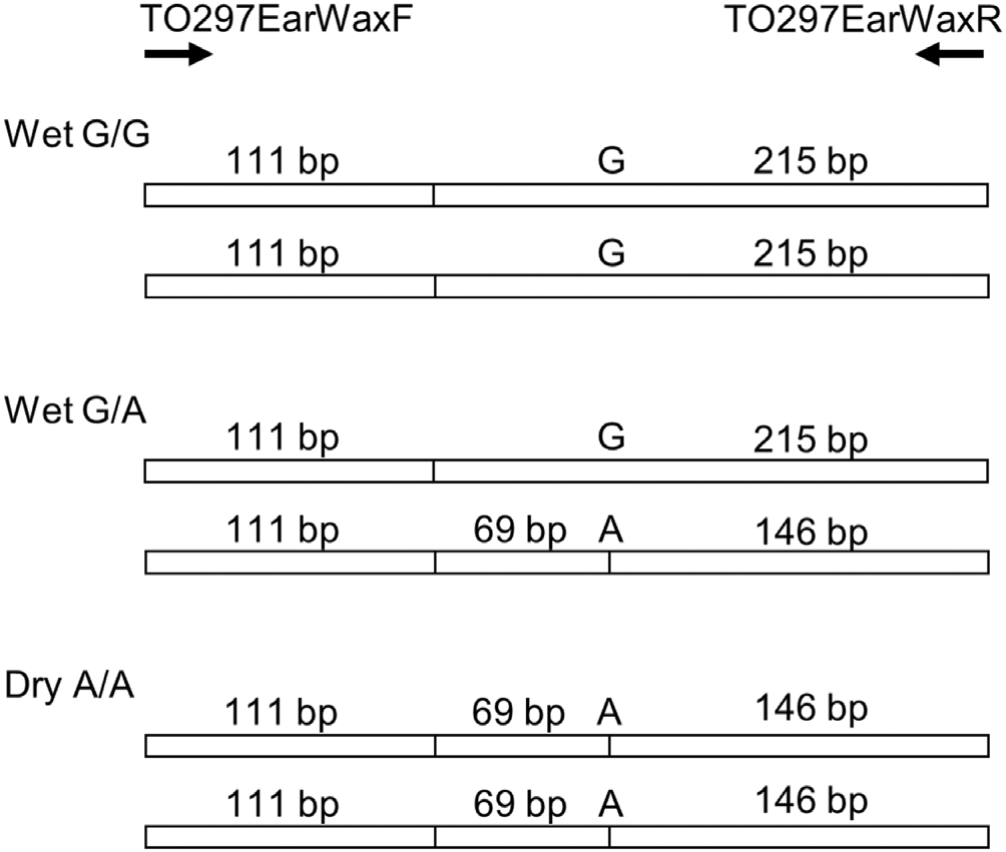
TO297EarWaxF and TO298EarWaxR. Schematic restriction enzyme recognized the site of the PCR product, including a single‐nucleotide polymorphism (SNP) site.

The 326‐bp PCR‐amplified product, when digested by DdeI, produces distinct band patterns for the A and G alleles. Specifically, digestion at rs17822931 generates a 111‐bp fragment common to both alleles. For the A allele, additional cleavage occurs, resulting in a 69‐bp fragment, whereas this additional cleavage is absent in the G allele. Consequently, the digestion pattern is visualized as a 111‐bp and 69‐bp band for the A allele and only a 111‐bp band for the G allele (Figures 1 and 2). Thus, the A or G genotype was easily visualized as a different band in the agarose gel using electrophoresis. Without purification after PCR, the DdeI restriction enzyme could digest the PCR product, including the PCR buffer. If the treatment of isolated DNA is inadequate (purification of genomic DNA does not amplify the target region, or the restriction enzyme performs poorly), the 111‐bp band is not detected. Therefore, the 111‐bp band was an excellent marker in this experiment.

### 3.2 The Recognition of Mendelian Inheritance Law

The students discussed and assessed individual results after the experiment. Consequently, they were able to understand molecular techniques and Mendelian law, including DNA isolation from buccal cells, PCR cloning, restriction enzyme digestion, electrophoresis in agarose gel, Mendelian trait, phenotype, genotype, dominant/recessive inheritance, allele frequency, Hardy–Weinberg principle, and genotype–phenotype association.

**FIGURE 3:**
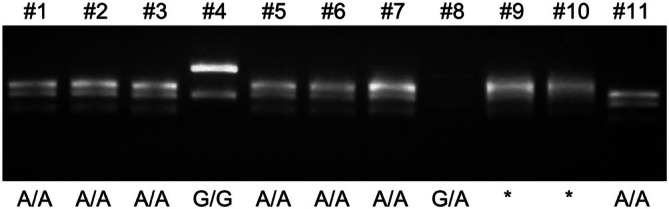
Actual electrophoresis photo performed by high school students, indicating failure to detect a clear band (*). Band #8 presents faintly, indicative of insufficient PCR amplification by one student.

For example, when 47 students participated in the course, 38, 4, and 1 had genotypes A/A, G/A, and G/G, respectively. The remaining four students failed to detect the genotype (Figure 3, Table 1). The G allele demonstrated a dominant phenotype [12]; therefore, the five students with genotypes G/G and G/A recognized the dominant phenotype and genotype of wet earwax, whereas the 38 students with genotype A/A recognized the recessive phenotype of dry earwax. The expected ratio of alleles G/G, G/A, and A/A was based on the Hardy–Weinberg principle, which calculates the actual frequency of the number of A and G alleles present (Table 1). The genetic data obtained through the experiment were analyzed, demonstrating the Mendelian inheritance of the ABCC11 gene's polymorphism. Students observed the direct correlation between their genotypes and phenotypes, and statistical analysis confirmed the expected distribution of traits according to the Hardy–Weinberg equilibrium.

**TABLE 1:** The result of allele frequency in one short course.

	Number	Ratio (%)
Genotype		
AA	38	88.4
GA	4	9.3
GG	1	2.3
Unknown	4	
Allele		
A	80	93.0
G	6	7.0

### 3.3 Computer‐Based Learning for Pedigree Drawing

In high school, students typically learn to construct simple pedigree charts, often based on Mendelian traits observed in plants or other organisms in biology class.

Following a lecture on the second day, the participants in this course create pedigrees using f‐tree, specialized software used in the clinical field, ensuring accuracy and efficiency. This activity highlights the significance of pedigrees in humans and their critical role in clinical applications.

**FIGURE 4:**
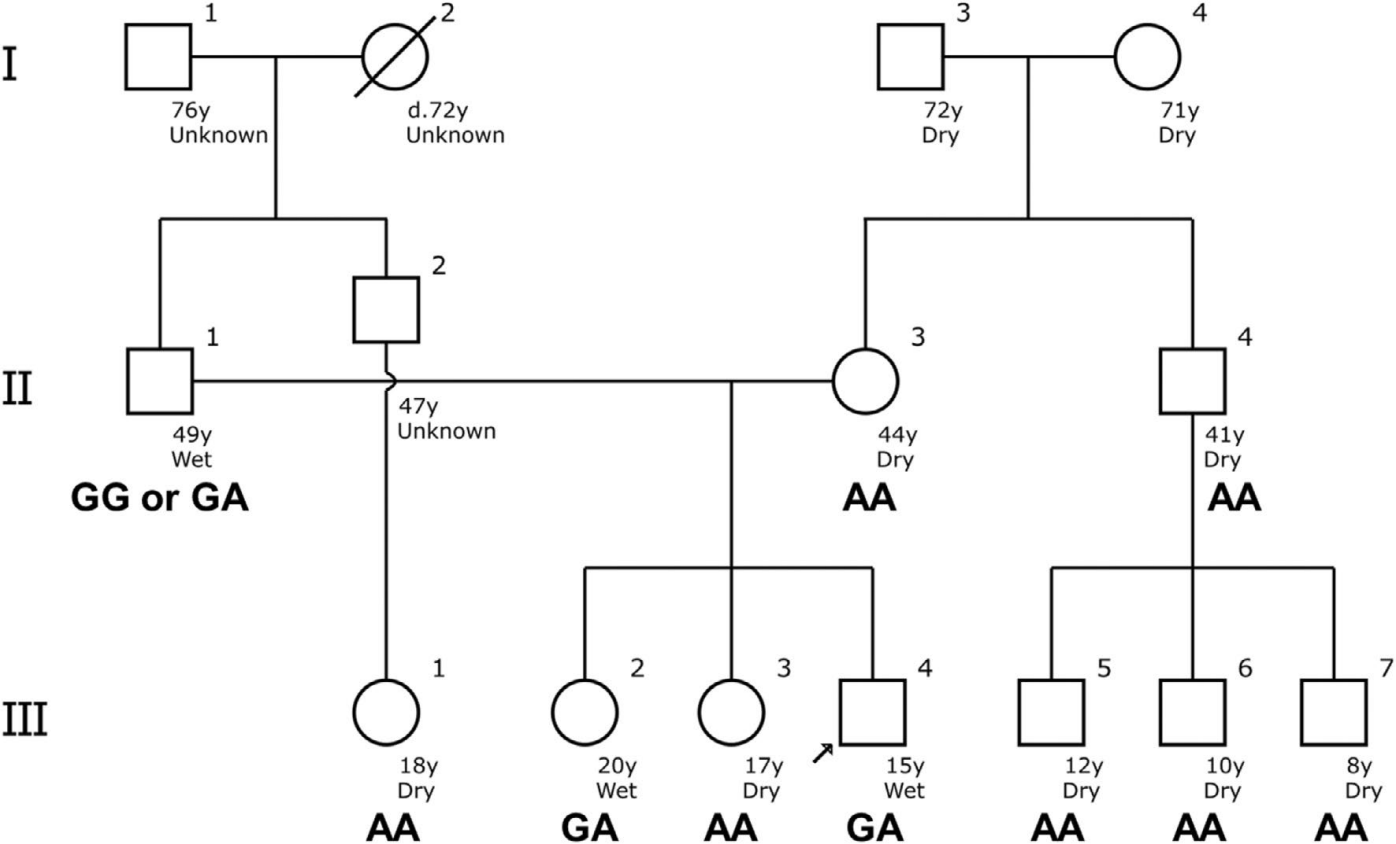
One pedigree chart created by a student using the f‐tree application. The f‐tree application supports English and Japanese inputs. The emphasized genotype is rs17822931 SNP, which was analyzed by the student.

Each student surveyed several phenotypes in individual family members. Some students tried to obtain family information, including earwax, the function of alcohol dehydrogenase in alcohol metabolism, carbohydrate intake, and hair color. These students were asked to input this information in the f‐tree application [17]. These traits from family members were automatically drawn in a pedigree chart (Figure 4). Most students generated pedigree charts that included the phenotypes of first‐degree family members and those of the second or third degree.

### 3.4 Questionnaire Results

Among the short course participants, 90 were very satisfied, 12 were satisfied, 16 were average satisfied, four were dissatisfied, zero were dissatisfied, and four did not respond (Table 2). In the survey after the course, more than 80% answered very satisfied or satisfied, and only 3% remained neutral.

**TABLE 2:** Evaluation of the genetic course in August 2019 and January 2020.

	#1	#2	#3	Total	Ratio (%)
Very satisfied	45	30	15	90	71.4
Satisfied	6	5	1	12	9.5
Average	8	4	4	16	12.7
Dissatisfied	2	2	0	4	3.2
Very dissatisfied	0	0	0	0	0
No answer	2	1	1	4	3.2
Total	63	42	21	126	

## 4 Discussion

This study leveraged the phenotype–genotype association of the ABCC11 polymorphism (rs17822931) to teach molecular genetics and Mendelian inheritance effectively. Conventionally, representative phenotypes of Mendelian inheritance in humans include the ability to roll one's tongue, unattached earlobes, dimples, freckles, straight hair, widow's peak, skin color, and cleft or smooth chins. While these traits are often used in genetics education, the mechanisms underlying many of them, particularly their determination by simple nucleotide variations, remain unclear. For example, genome‐wide association studies have revealed that the earlobe attachment phenotype is associated with multiple loci, including EDAR, SP5, MRPS22, ADGRG6 (GPR126), KIAA1217, and PAX9 [18]. This highlights the complexity of polygenic inheritance and underscores the challenges of teaching that emphasizes such traits as being purely Mendelian. Similarly, while straight and curly hair are easily distinguishable and appear as all‐or‐nothing phenotypes, multiple SNPs associated with a single gene contribute cumulatively to these traits, making their molecular analysis more complex [19].

In contrast, the ABCC11 polymorphism offers a straightforward model of a single‐gene trait [12]. The G‐to‐A substitution at rs17822931 in the first transmembrane domain of ABCC11 results in a loss of transport function, leading to the dry earwax phenotype [12]. The A allele is recessive, while the G allele is dominant, making this a clear and observable example of Mendelian inheritance. By calculating allele frequencies using the Hardy–Weinberg principle and conducting hands‐on experiments, students gained insights into key genetic concepts such as homozygosity and heterozygosity.

Our methodology, which included PCR amplification of the region around rs17822931 followed by DdeI restriction enzyme digestion, provided a hands‐on approach to molecular genetics. This method eliminated the need for PCR product purification and allowed students to visualize genotype differences through electrophoresis. Unlike real‐time PCR, which requires specialized equipment and software, the DdeI method is accessible and can be reliably deployed in high school settings. Additionally, the use of earwax phenotypes was integrated into family pedigree construction using the f‐tree software. Students created standardized pedigree charts, linking phenotypic and genotypic data, which further enhanced their understanding of inheritance patterns and also served to introduce them to genetic tools used in clinical settings [17]. Moreover, the pedigree chart created by the students complies with the international standardized human pedigree nomenclature [20, 21].

Our program's comprehensive features and advantages lie in its ability to enable even beginners in experimental science to almost certainly determine their genotype for a particular trait using molecular genetics techniques. Additionally, participants can construct their family pedigrees through computational analyses, as wet‐ and dry‐lab experiments progress concurrently throughout the two‐day program. This is facilitated by simplifying basic molecular biology procedures and including designated time for pedigree creation, ensuring flexibility in time management. This setup allowed us to monitor each step of the students' operations in real time and provide immediate recovery and support when necessary. Through this structured program, all students were able to successfully obtain genotyping results, which we believe have significantly deepened their understanding of the subject matter. Nevertheless, one or two students encountered DNA extraction or PCR amplification difficulties in each session. These challenges were not due to conceptual misunderstandings, but rather technical issues, most commonly including pipetting errors, such as inaccurate volume handling during reagent addition. This highlights the critical importance of mastering basic experimental techniques in molecular biology. While scientific evaluation assumes accurate and reproducible experimental procedures, students must learn that data reliability fundamentally depends on the precision of such technical execution. This realization not only cultivates technical discipline but also fosters a deeper understanding of the principles of experimental science.

This experimental setup not only allows for the direct experience of Mendelian inheritance patterns but also provides an opportunity to study the molecular biological functions of ABCC11. The ABCC11 gene encodes a protein that belongs to the MRP subfamily, characterized by two ATP‐binding domains and 12 transmembrane domains [12]. One of its primary functions is to mediate the transport of substances across the cell membrane for secretion into the extracellular space [12]. Notably, a single‐nucleotide polymorphism (SNP), specifically the G‐to‐A transition at rs17822931, located within the first transmembrane domain of MRP8, leads to a loss of transport function. This functional loss underlies the dry earwax phenotype, serving as a determining genetic factor [12, 16, 22, 23]. This study can also provide insights into biologically active chemicals' intracellular and extracellular transport mechanisms.

Furthermore, this framework not only emphasizes scientific aspects but also provides students with a basis for discussion of historical patterns of migration and the movement of specific populations throughout history. The genotype frequency of the A and G alleles at rs17822931 has been analyzed in various ethnicities and races in modern and ancient civilizations [13, 18, 19, 20]. The results from these studies suggest that the A allele originated around Mongolia and expanded dominantly to East Asia [12]. A study performed by a Japanese nationwide consortium of Super Science High Schools demonstrated that local accumulation of the A allele was observed in Japan and globally in the localization of A or G alleles [16]. Previous studies have suggested that the difference in A or G allele frequency depends on the different ancient civilizations that inhabited the Japanese Islands. A common hypothesis is that the Japanese population originated from a mixture of two populations, the Jomon and Yayoi [22, 23]. The ancient Jomon population, which lived on the whole island of Japan, had wet earwax with the G/G genotype [22, 23]. Approximately 3000–1800 years ago, the Yayoi landed on Japanese islands as new immigrants from the Korean Peninsula or the Chinese mainland. The frequency of the A allele localization indicated an invasion by the Yayoi people since the Chinese and Korean peoples had a high frequency of the A allele [22, 23, 24]. These narrations of old East Asian migration are taught in high school history classes. Notably, Kyushu Island, close to the Korean Peninsula or the Chinese mainland, and the Setouchi and Kansai regions have a high frequency of the A allele [16]. The families of the students who attended the short course are residents of Hokkaido, located on the northernmost Japanese Island, and have a relatively new history of migration from the rest of the Japanese region. The analyzed ratio of the A or G allele in our short course reflects the average Japanese frequency of the A or G allele. Additionally, the students examined the frequency of the G or A allele in European and East Asian regions and observed that the frequency of the A allele is higher in the East than in the West [12]. This observation may be explained by the prominent events in world history that occurred in the European and East Asian regions. Putative evidence shown to the students includes the expeditions by Mongolians that happened around the thirteenth century.

In addition to earwax phenotypes, SNP detection experiments related to athletic performance and the ACTN3 gene [25], bitter taste perception, and the TAS2R38 gene [26], alcohol metabolism‐associated genes [27], blood type and the ABO gene [23, 28], as well as caffeine sensitivity and the CYP1A2 gene [29] may also capture students' interest. Furthermore, such experiments can potentially be conducted at the high school level. However, the module we adopted, which focuses on the SNP and phenotype of ABCC11, offers distinct advantages. The global frequencies of the A and G alleles have been elucidated, and the molecular biological mechanisms underlying their phenotypic expression have been established. This approach uniquely integrates science with historical perspectives, offering an interdisciplinary educational benefit.

While the course was highly effective, with 81% of students expressing satisfaction and 71% reporting high satisfaction, some limitations remain. The small sample size and specific educational setting limit generalizability, and the focus of the course on Mendelian traits does not address the full complexity of polygenic and environmental influences. Additionally, the short duration of the course restricted in‐depth exploration, and self‐reported feedback may not fully capture the learning outcomes.

Despite these challenges, the module successfully provided students with a practical introduction to molecular genetics, human genetic variation, and historical insights, offering a reproducible framework for teaching genetics at the high school level.

## Author Contributions

Methodology: T.O., R.T., T.A., and T.T. Writing: T.O. and T.T. Investigation: A.Y., D.P., S.G., T.K., and Y.K. Writing – review and editing: T.T.

## Ethics Statement

The Institutional Ethics Board of the Human Genome Department (#20‐Hg‐003) at Health Sciences University of Hokkaido approved this study.

## Consent

Before starting the genetic course, written informed consent was obtained from all students' parents and attendees.

## Conflicts of Interest

The authors declare no conflicts of interest.

## Supporting information


**Data S1:** bmb70010‐sup‐0001‐Supinfo.

## Data Availability

The datasets used and analyzed in the current study are available from the corresponding author upon reasonable request. However, the data are not publicly available due to privacy protection concerns.
