# Supplementary material for: ABCC11 Earwax Trait and Genotype Are Suitable Tools for Introductory Labs to Learn Genetics and Molecular Techniques
Source: Biochem Mol Biol Educ. 2025 Aug 13;53(6):603–9. doi: 10.1002/bmb.70010 (PMC12671274; doi:10.1002/bmb.70010)
Supplement: Supplementary file 1 — Data S1: bmb70010‐sup‐0001‐Supinfo. [file BMB-53-603-s001.docx]

**A Simple Guide to Genotyping Earwax for Students**

**A. Collect oral mucosal cells with a swab.**

1) Write your name on the lid and side of the 2 ml tube.

2) Rub your inner cheeks 5–6 times firmly with a swab.

3) Push the end of the handle to remove the swab.

**B. Dissolving the cell membrane, nuclear membrane, and protein.**

1) Add 20 μl Proteinase K and 600 μl ATL to the sample, close the lid, and mix well.

Caution! ATL and AL, AW 1, should absolutely not come into contact with the eyes or the skin!

2) Incubate at 56 °C for 1 hour. (Mix it several times in the middle.)

3) 600 μl AL to the sample, close the lid, and mix well.

4) Incubate at 70 °C for 1 hour. (Mix it several times in the middle.)

5) Centrifuge for several seconds with a mini centrifuge.

6) Add 300 μl ethanol to the sample, close the lid, and mix well.

Completion of the lysate.

**C. Tangle the DNA to the filter.**

1) Write your name on the filter tube cap.

2) Put 700 μl of the solution (total volume about 1.5 ml) into the tube on the filter. Avoid swabs at this time.

3) Centrifuge for 30 seconds in a centrifuge.

4) Replace the liquid collection tube with a new collection tube. (At this stage, DNA is present, tangled in the filter.)

5) The remaining solution is put into the tube on the filter. Avoid swabs at this time.

6) Centrifuge for 30 seconds in a centrifuge.

7) Replace the liquid collection tube with a new collection tube.

**D. While entangling the DNA on the filter and washing it,**

1) Place 1500 μl of AW1 into a filter tube and centrifuge for 30 seconds in a centrifuge.

2) Replace the liquid collection tube with a new collection tube.

3) Place 1500 μl of AW2 into a filter tube and centrifuge for 30 seconds in a centrifuge.

4) Replace the liquid collection tube with a new collection tube.

5) Centrifuge at maximum speed for 3 minutes in a centrifuge to dry the filter membrane.

**E. Remove DNA from the filter and extract.**

1) Write your name on the lid and side of the 1.5 ml tube.

2) Place the washed filter tube on the tube.

3) Put 20 μl of distilled water into the center of the membrane.

4) Allow to stand for 1 minute and then centrifuge for 1 minute at maximum speed. Discard the filter tube.

DNA extraction completed.

**F. Increase DNA by PCR.**

1) Write your name on the side of the PCR tube.

2) Mix 5 μl of extracted DNA into 45 μl of PCR mix.

3) Place in the thermal cycler and start the program.

**G. Cut the DNA product with a restriction enzyme.**

1) Write your name on the 1.5 ml tube cap.

2) Mix 20 μl DNA product into the restriction enzyme mix (total volume 40 μl).

3) Incubate at 37 °C for 1 hour or more.

**H. Check the cleavage pattern with restriction enzymes on agarose gel.**

1) Mix 20 μl of the DNA cut with the restriction enzyme with the dye and place it in the hole of the agarose gel.

2) Irradiated with UV, see the cutting pattern. (Instructor)
